# Effect of Countersample Coatings on the Friction Behaviour of DC01 Steel Sheets in Bending-under-Tension Friction Tests

**DOI:** 10.3390/ma17153631

**Published:** 2024-07-23

**Authors:** Tomasz Trzepieciński, Krzysztof Szwajka, Marek Szewczyk, Marek Barlak, Joanna Zielińska-Szwajka

**Affiliations:** 1 Department of Manufacturing Processes and Production Engineering, Faculty of Mechanical Engineering and Aeronautics, Rzeszow University of Technology, al. Powstańców Warszawy 8, 35-959 Rzeszów, Poland; 2 Department of Integrated Design and Tribology Systems, Faculty of Mechanics and Technology, Rzeszow University of Technology, ul. Kwiatkowskiego 4, 37-450 Stalowa Wola, Poland; kszwajka@prz.edu.pl (K.S.); m.szewczyk@prz.edu.pl (M.S.); 3 Plasma/Ion Beam Technology Division, Material Physics Department, National Centre for Nuclear Research Świerk, 7 Sołtana St., 05-400 Otwock, Poland; marek.barlak@ncbj.gov.pl; 4 Department of Component Manufacturing and Production Organization, Faculty of Mechanics and Technology, Rzeszow University of Technology, ul. Kwiatkowskiego 4, 37-450 Stalowa Wola, Poland; j.zielinska@prz.edu.pl

**Keywords:** coefficient of friction, bending under tension, deep drawing, friction conditions, sheet metal forming

## Abstract

The aim of this article is to provide an analysis of the influence of the type of hard anti-wear coatings on the friction behaviour of DC01 deep-drawing steel sheets. DC01 steel sheets exhibit high formability, and they are widely used in sheet metal forming operations. The tribological properties of the tool surface, especially the coating used, determine the friction conditions in sheet metal forming. In order to carry out the research, this study developed and manufactured a special bending-under-tension (BUT) friction tribometer that models the friction phenomenon on the rounded edges of tools in the deep-drawing process. The rationale for building the tribotester was that there are no commercial tribotesters available that can be used to model the phenomenon of friction on the rounded edges of tools in sheet forming processes. The influence of the type of coating and sheet deformation on the coefficient of friction (CoF) and the change in the topography of the sheet surface were analysed. Countersamples with surfaces prepared using titanium + nitrogen ion implantation, nitrogen ion implantation and electron beam remelting were tested. The tests were carried out in conditions of dry friction and lubrication with oils with different kinematic viscosities. Under dry friction conditions, a clear increase in the CoF value, with the elongation of the samples for all analysed types of countersamples, was observed. Under lubricated conditions, the uncoated countersample showed the most favourable friction conditions. Furthermore, oil with a lower viscosity provided more favourable conditions for reducing the coefficient of friction. Within the entire range of sample elongation, the most favourable conditions for reducing the CoF were provided by uncoated samples and lubrication with S100+ oil. During the friction process, the average roughness decreased as a result of flattening the phenomenon. Under dry friction conditions, the value of the Sa parameter during the BUT test decreased by 20.3–30.2%, depending on the type of countersample. As a result of the friction process, the kurtosis and skewness increased and decreased, respectively, compared to as-received sheet metal.

## 1. Introduction

Friction is generally considered undesirable in sheet metal forming processes. It increases forming forces, deteriorates the surface quality of drawpieces and increases the wear on tools [1]. Therefore, since the beginning of metal forming, efforts have been made to minimise resistance to friction by using appropriate lubricants [2], self-lubricating coatings [3], pressure-assisted lubrication [4] and hard wear-resistant coatings [5]. Friction depends on many technological factors (e.g., static or dynamic character of contact [6,7], sliding speed [8,9], contact pressure [10,11]), material factors (chemical affinity of rubbing metals [12], surface roughness [13,14], surface texture [15,16], contact area [17], hardness of the tool and sheet material [18,19], type of protective coating [20,21]) and working conditions (temperature [22,23], physicochemical properties of the lubricant [24,25]). A quantitative parameter describing the phenomenon of friction is the coefficient of friction (CoF). According to the basic Amontons–Coulomb hypothesis, the CoF is the ratio of the frictional force to the normal force pressing the two surfaces together [26].

In sheet metal forming processes, a hard tool comes into contact with a sheet material of much lower strength [4,27]. In such conditions, the friction phenomenon is constantly evolving due to the plastic deformation of the sheet and changes in surface topography [28,29]. Friction is accompanied by flattening, roughening and work-hardening phenomena that change the properties of the sheet [30].

To recognize the phenomenon of friction in individual zones of the deep-drawn sheet, that is, in the flange zone, on the edges of the die and punch and on the side wall surface, it is necessary for the forming process technology to follow the correct design. Over the years, many independent tribological tests have been developed to determine the CoF in the above-mentioned zones of formed sheet metal [31,32]. In a bending-under-tension (BUT) test, the strip specimen is wrapped around a cylindrical countersample (wrap angle 90°) and is stretched until fracture. In this way, it is possible to determine the evolution of the value of the CoF during the forming process. Investigations aimed at determining the CoF on the punch edge using BUT tests are not widespread in the available literature compared to the common use of strip drawing tests. The BUT test has been developed by Littlewood and Wallace [33]. Uko et al. [34] investigated the strain distribution of high-strength low-alloy steel sheets in the BUT test. It was found that, toward the end of the BUT process, the bending strains at both the outer and inner surfaces of samples are tensile. These strains increase at an approximately equal rate. Kotchman et al. [35] determined the frictional behaviour of drawing-quality steel sheets using a BUT test under varying die radii and sliding speeds. The orthogonal array technique was used to ascertain the relations between friction process parameters and friction forces. They found that countersample materials play an important role in determining the CoF. Vallace and Matlock [36] applied the BUT friction test to investigate the friction behaviour of zinc-coated steel sheets. They compared the different equations used to calculate the CoF and concluded that the determination of the CoF based on an energy analysis is equivalent to the solution based on a force balance. Andreasen et al. [37] developed the device for a BUT test with a direct friction measurement and tool preheating system. They observed a significant effect of contact pressure and lubricant type on the limits of lubrication. Lemu et al. [38] investigated the influence of the amount of plastic deformation of deep-drawing quality steel sheets on the CoF in dry and lubricated conditions using a BUT friction test. Both FE-based numerical modelling and experiments were carried out. It was found that a lubricated contact condition reduces the CoF to a higher degree for countersamples with higher surface roughness. Folle and Schaeffer [39] evaluated the contact pressure between the sheet and the countersample in a BUT test by using pressure-sensitive film. The results revealed that the measurement of vertical force on the cylindrical countersample is closer to the real values than the analytical formulas used so far for this. Ceron et al. [40] analysed the temperature variation in BUT tests through a combined finite element-based numerical–experimental approach. A newly built tribometer allows for the modelling of the distribution of temperature resulting from the 1000 strokes emulating the temperature increase in production. The proposed methodology accurately predicts the interface temperature in the tribometer. Wiklund et al. [41] proposed a friction model that considered the effect of bulk plastic strains on the real area of contact. A comparison of the developed model with the experimental results of the BUT friction test has shown the potential of improving the numerical modelling of the deep-drawing process in comparison to the use of Coulomb’s friction model. Numerical modelling of the material flow in the BUT test has been numerically analysed by Trzepieciński and Lemu [42]. It was found that contact pressure varies along the width and length of the strip material in contact with the countersample surface. A similar conclusion has been found by Sniekers et al. [43] and Kim et al. [44]. Pressure in the contact interface is non-uniform, and Nielsen et al. [45] proposed modified formulae for the determination of the CoF in the qualitative analysis of the tribological behaviour of metallic sheets in a BUT test.

In this article, the BUT test was used to analyse the phenomenon of friction at the rounded edge of a stamping die. This area is critical in sheet metal forming due to the large deformations of the sheet metal and the risk of premature cracking of the sheet metal. As the BUT test simulates the friction conditions on the edge of stamping dies, one of the grades of deep-drawing steel sheets (DC01) was selected as the test material. DC01 steel sheets exhibit high formability, and they are widely used in sheet metal forming operations. The most frequently used tribological test in sheet metal forming processes is the strip drawing test. Meanwhile, the use of the BUT test is very limited in the literature. This test requires a special tribotester. However, there are no commercial devices available for modelling friction using the BUT test. For this reason, the phenomenon of friction in sheet metal forming is commonly tested using a simple strip drawing test. However, this test does not fully reflect the complex contact phenomena occurring on the rounded edges of stamping dies. The results presented in this article are determined using the BUT friction tester developed and manufactured by the authors. This article examines the influence of the type of tool coating on the value of force parameters and, therefore, on the value of the CoF. The influence of friction on the temperature change in the contact zone was also determined, which, to the best of our knowledge, has not yet been tested under the conditions of sheet metal deformation in the BUT test.

## 2. Test Material and Methods

### 2.1. Material

The research materials were DC01 steel sheets with a thickness of 0.8 mm. DC01 steel sheets exhibit high formability, and they are widely used in sheet metal forming operations. So, it is a very good choice to test the influence of the type of hard anti-wear coatings on the friction behaviours in sheet metal forming. The requirements for the chemical composition of the sheet material, in accordance with EN 10130:2009 [46], are presented in Table 1.

The basic mechanical properties of sheets, determined using a uniaxial tensile test in accordance with EN ISO 6892-1:2020 [47], are presented in Table 2. The tests were carried out using a Zwick/Roell Z100 (Zwick Roell Group, Ulm, Germany) testing machine. Samples cut along and transversely to the direction of sheet rolling were stretched. The test samples had the shape of sheet metal strips 200.0 mm long and 25.0 mm wide, in accordance with EN ISO 6892-1:2020 [47]. Three repetitions were used for each direction of sheet cutting, thus determining the average values of mechanical parameters.

**Figure 1:**
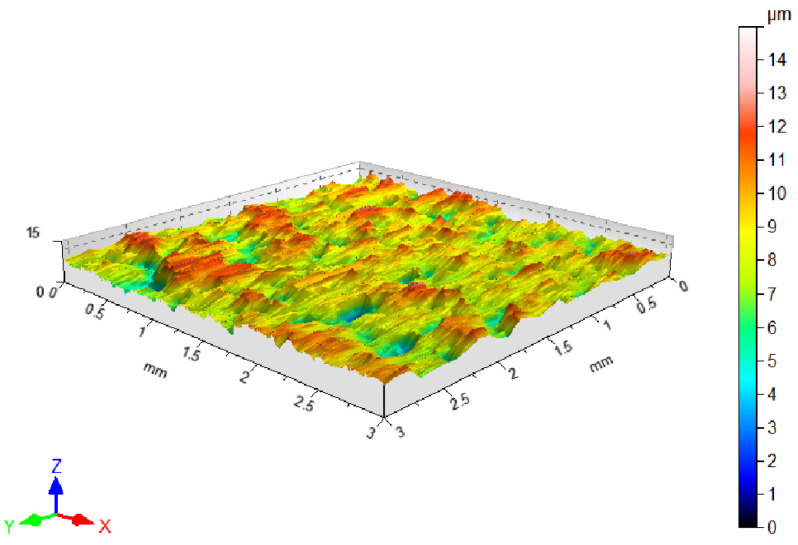
Surface topography of DC01 sheet metal.

The surface topographies of the DC01 steel sheet in its as-received state (Figure 1) and after friction tests were measured using a T8000RC stationary profilometer from Jenoptik AG (Jena, Germany). The maximum height of the roughness profile was approximately 14 μm (Figure 1). The surface topography was characterized by uneven distribution of surface asperities. Basic 3D surface roughness parameters (Table 3) were determined according to standard ISO 25178-2 [48]. Values of average roughness (Sa) and root-mean-square roughness parameter (Sq) were 1.28 μm and 1.63 μm, respectively. The maximum pit depth (Sv) was 8.39 μm, while the highest peak of the surface (Sp) was 5.09 μm. The 10-point peak–valley surface roughness (Sz) is the sum of the parameters Sv and Sp. The kurtosis value of 3.63 indicates that the bearing area curve is bell-shaped and has relatively many high peaks and shallow valleys. A negative skewness value (Ssk = −0.407) characterizes a surface with shallow deep valleys. The morphology of the countersample surfaces was observed using Keyence VHX-7100 (Osaka, Japan) optical microscope (OM). The hardness of the DC01 sheet metal, calculated on the basis of five measurements, was 107 HV. The hardness measurement of the sheets was carried out using the Ernst Dynatest SCX (Lamone, Switzerland) hardness tester.

### 2.2. Experimental

**Figure 2:**
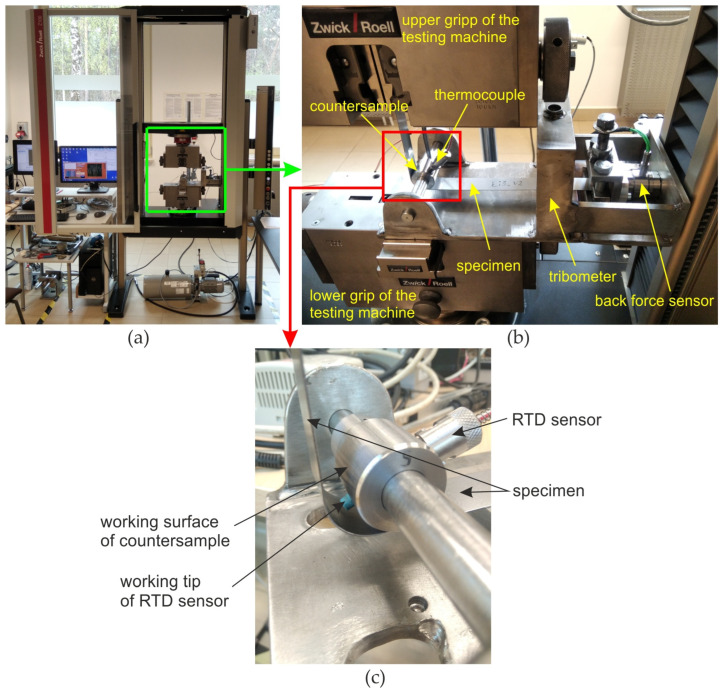
(**a**) Test stand, (**b**) specialized tribometer for BUT test and (**c**) RTD sensor location.

For friction tests, a specialised tribometer (Figure 2) was designed and manufactured to perform the BUT test. This tribological test simulates friction at the edges of tools in sheet metal forming. The tribometer consists of a holder integrated with a Kistler^®^ type 9345B (Kistler, Winterthur, Switzerland) force sensor, in which one of the ends of the specimen is mounted. Sheet metal strips 25 mm wide and 400 mm long were tested. The specimens were cut along the rolling direction of the sheet metal. The other end of the sample was mounted in the upper grip of a Zwick/Roell Z100 (Zwick Roell Group, Ulm, Germany) testing machine. The sample was wrapped around a cylindrical countersample. The wrap angle (Θ) was 90°. The temperature in contact zone was measured using resistance temperature detector (RTD) 745691-02 (National Instruments, Austin, TX, USA) which was sealed in an alumina tube with three teflon-coated leads. The RTD sensor was placed in a hole drilled in the countersample (Figure 2c) and made direct contact with the surface of the sheet metal. In Figure 2c, the RTD sensor is disclosed so that its tip can be observed. Under friction test, the specimen and working tip of RTD sensor adheres to the working surface of countersample. Temperature was recorded using the National Instruments NI cDAQ-9132 station, the NI 9216 temperature measurement module and the NI SignalExpress 2015 program.

**Figure 3:**
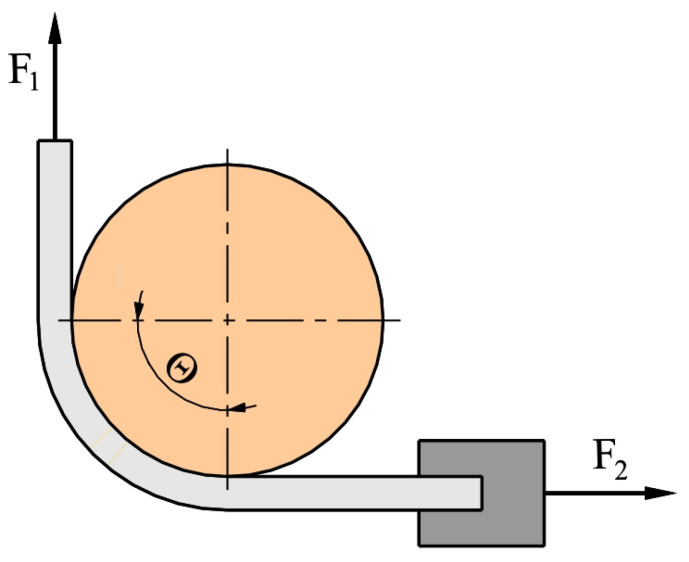
Schematic of BUT test.

During the test, the movement of the upper grip of testing machine at speed of 30 mm/min was initiated and the test continued until the sheet metal strip broke. During the test, the front tension force (F_1_) (Figure 3) was recorded by the measuring system of the tensile testing machine. Back tension force (F_2_) was recorded by the Kistler^®^ type 9345B force sensor. The back tension force signal was recorded using a personal computer via a Kistler type 5073 charge amplifier and terminal block (National Instruments type BNC2110). Both front tension force and back tension force were recorded with a frequency of 100 Hz. The occurrence of friction between the surfaces of the sheet metal strip and the countersample caused the front tension force to be greater than the back tension force. The value of the CoF for the wrap angle (Θ = 90°) was determined from the equation [49]:

(1) $$\mathrm{CoF}=\frac{2\;}{\pi }\ln\frac{F_{1}}{F_{2}}$$

where F_1_ is front tension force recorded by the measuring system of the tensile testing Zwick/Roell Z100 machine and F_2_ is back tension force recorded by the Kistler^®^ type 9345B force sensor. Measuring accuracy of F_1_ force was +/−0.2% (manufacturer’s data—Zwick Roell Group, Ulm, Germany). The sensitivity of 9345B F_2_ force sensor was about 3.7 pC/N (manufacturer’s data - National Instruments, Austin, TX, USA).

The sheet metal strips were tested in a friction pair with four countersamples made of a chromium-alloyed 145Cr6 tool steel (1.2063). This material was used as a substrate to produce various anti-wear coatings (Table 4). For comparison, uncoated samples were also tested. Table 5 also shows the hardness of the tested materials determined using the Ernst Dynatest SCX hardness tester in accordance with the ISO 6507-1 [50] standard with five repetitions. The basic 2D surface roughness parameters characterizing the surfaces of the countersamples (Table 5) were determined according to standard ISO 4287 [51]. Due to the high smoothness of some countersamples (C-Ti-N, C-N and C-U) and their rounded surface (R = 15 mm), accurate measurement of 3D roughness parameters using our contact device was not possible. So, these parameters were measured on the surface along the axis of all the countersamples. The surface roughness of the countersamples was measured with five repetitions.

Friction tests were performed at a temperature of 20 °C under dry friction and lubricated conditions. An Ostwald viscometer was used to determine kinematic viscosity (η_k_) of lubricants. The S100+ (η_k_ = 360 mm^2^/s) and S300 (η_k_ = 1135 mm^2^/s) oils for deep-drawing operations were used as lubricants. The tested oils were produced from deeply refined mineral oils and vegetable oil. They contain anti-corrosion additives and additives that increase their lubricating properties. Greases with significantly different viscosities were selected to test the analysed coatings over a wide range of input parameter variations. In sheet metal forming, viscosity is a fundamental parameter characterizing liquid lubricants. After each test, the countersamples were cleaned with acetone to remove any contaminants. The oil was applied to the specimen surface with a soft brush [52]. Then, the specimen was stood up for 60 s so that the excess of oil flowed away by gravity.

## 3. Results and Discussion

### 3.1. Coefficient of Friction

**Figure 4:**
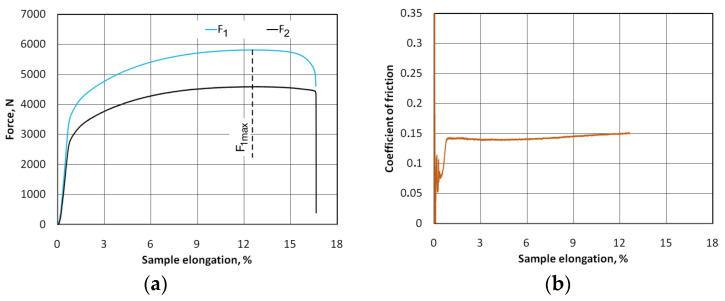
(**a**) Effect of sample elongation on the variation of test forces and (**b**) resultant CoF for C-Ti-N countersample (lubrication with S100+ oil).

**Figure 5:**
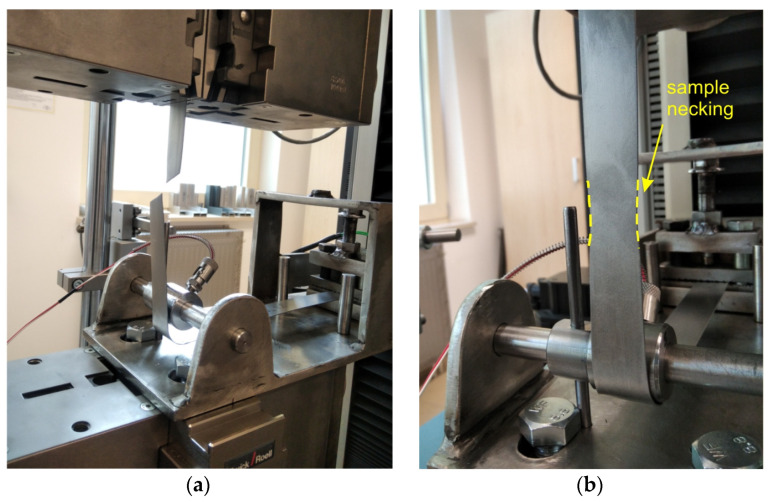
(**a**) Strip sample after BUT friction test and (**b**) sample necking.

As a result of BUT friction tests, the variations of the front tension force and back tension force were obtained for each of the analysed countersample configurations and friction conditions. Based on these, according to Equation (1), the variation of changes in the CoF, with the elongation of the samples, was determined. The results for the C-Ti-N countersample configuration and dry friction conditions are shown in Figure 4. Based on the equation proposed by Wihlborg and Gunnarsson [53], the value of average unit pressure in the analysed range of sample elongation until failure was between 0 and approximately 14 MPa (at sample elongation of 12%). The value of the maximum contact pressure (14 MPa) takes into account the change in the width of the sample as a result of the stretching process. In the friction tests, the width of the samples in the contact zone decreased by approximately 1 mm compared to the initial width (25 mm). Due to the friction occurring between the countersample and sheet metal, the front tension force showed higher values than the back tension force. Both the front tension and back tension forces graphs are stable without fluctuations (Figure 4). This proves the stability of the friction conditions. In addition, high-class professional commercial measurement sensors and a Zwick/Roell testing machine with a measuring accuracy of F_1_ force +/−0.2% (manufacturer’s data - Zwick Roell Group, Ulm, Germany) were used in the experiments. The instability of force measurement began when the F_1_ force reached its maximum value. This is related to the sample necking that occurred. However, the force values after exceeding the maximum force were not taken into account when determining the CoF. When the front tension force (F_1_) reached its maximum value, the necking was localized in the sample zone affected by the front tension force (Figure 5a). Under these conditions, the sample strain state changed from uniaxial to triaxial, and further deformation occurred at the point where the sample necking occurred (Figure 5b). At the same time, after exceeding the maximum value of the front tension force, the relative displacement of the sample against the surface of the countersample did not occur. Therefore, a physically valid value of the coefficient of friction can only be determined up to the maximum force (F_1max_) (Figure 4).

**Figure 6:**
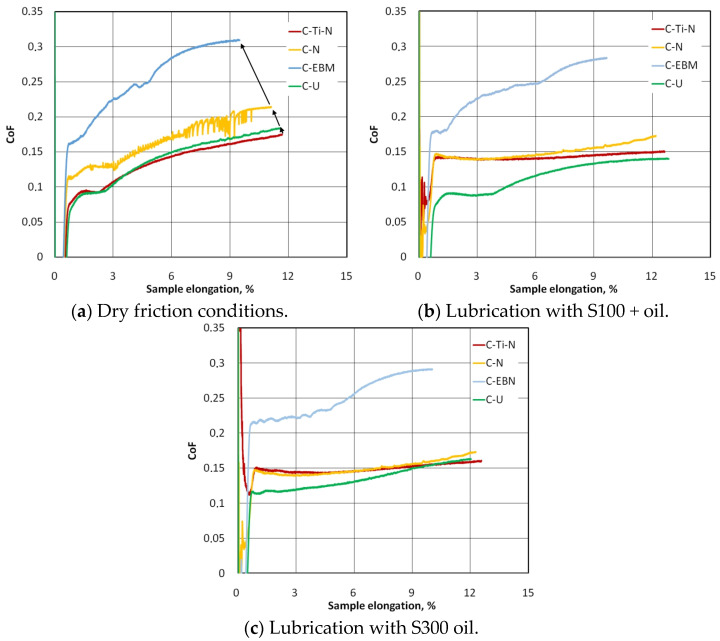
Evolution of the coefficient of friction in BUT friction test under (**a**) dry friction conditions and lubrication of the contact interface with (**b**) S100+ oil and (**c**) S300 oil. C-Ti-N is titanium- and nitrogen-ion implanted countersample, C-N is nitrogen-ion implanted countersample, C-EBM is electron-beam-melted countersample and C-U is uncoated countersample.

Figure 6 shows the change in the value of the coefficient of friction with the increase in the elongation of the strip samples. Under dry friction conditions, a clear increase in the CoF value, with the elongation of the samples for all analysed types of countersamples, was observed. Dry friction causes intensive braking of sheet material movement with increasing strip sample elongation. Within the considered sample elongation range, contact pressures increase simultaneously until the front tensile force reaches its maximum value (Figure 4). As the sample elongation increases, the ratio between front and back tension forces increases and, as a result (Equation (1)), the CoF increases (Figure 6a).

Increased friction in the contact zone between the sheet metal and the countersample limits the elongation of the material, and the greater the friction, the lower the elongation of the samples (Figure 6a). It should be noted that this conclusion applies to the range of sample elongation from the beginning of the test until the front tension force reaches the maximum value (F_1max_). The CoF determined for uncoated counterspecimens (C-U curve in Figure 6a) and titanium- and nitrogen-ion implanted countersamples (C-Ti-N curve in Figure 6a) are similar. Under conditions of dry friction, the highest value of the coefficient of friction, up to approximately 0.31, was recorded for the C-EBM countersample. The conclusion above can be generalized to other friction conditions involving S100+ (Figure 6b) and S300 (Figure 6c) lubricants. This can be directly related to the much higher average roughness of this countersample compared to the other countersamples (Table 6). The hardness of the materials of all countersamples (Table 5) was higher than that of the material of the test sheet metals (107 HV). So, the surface roughness of the countersample has a significant impact on the change in the topography of the softer sheet as a result of the flattening and ploughing mechanisms [54,55].

Except for the electron-beam-melted countersample (C-EBM), under lubricated conditions, the friction coefficient was more stable in the S100+ (Figure 6b) and S300 (Figure 6c) oil lubrication conditions, and the uncoated countersample showed the most favourable friction conditions. Under dry friction conditions (Figure 6a), the coefficient of friction for the uncoated countersample was only slightly larger than that for the titanium- and nitrogen-ion implanted countersample (C-Ti-N). The lubricant facilitated the movement of the sheet over the surface of the countersample, and under these conditions, the ratio of front tension to back tension forces was more stable than during dry friction conditions. This is according to Equation (1) which ensured a stable CoF value during the test (C-Ti-N in Figure 6b,c).

In the case of electron-beam-melted countersamples, lubricating the surface with S100+ (Figure 6b) and S300 (Figure 6c) oils resulted in a reduction of the maximum CoF value by approximately 9.6% and 7.1%, respectively. The character of the changes in the CoF is similar for all friction conditions because, as the samples lengthened, the value of the force parameters of the friction process increased (Figure 4a) as a result of the work-hardening phenomenon. In the lubricated conditions, in the initial range of sample elongation, a certain stabilization of changes in the CoF value was observed (C-Ti-N and C-N in Figure 6b,c), and only from the elongation value of approximately 6% did the CoF value began to increase. This effect can be attributed to the formation of a ‘lubricant cushion’ which limited the metallic contact of the surface asperities. With an increasing value of the contact pressure of the sample on the countersample, the beneficial effect of the lubricating cushion is gradually reduced by two mechanisms of the mechanical impact of the surface asperities (flattening and ploughing [56,57]). In the case of uncoated countersamples, S100+ and S300 oils reduced the maximum CoF value by approximately 21.0% and 9.4%, respectively. Lubrication causes the CoF value determined with the C-Ti-N, C-N and C-U countersamples (Figure 6b,c) to be more uniform throughout the test compared to dry friction conditions (Figure 6a).

**Figure 7:**
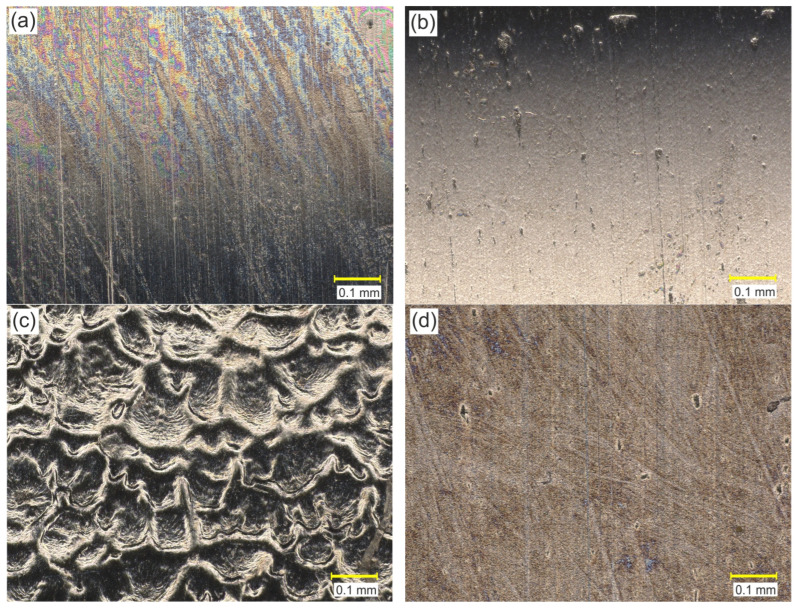
OM morphology of the countersample surfaces: (**a**) C-Ti-N, (**b**) C-N, (**c**) C-EBM and (**d**) C-U.

The morphology of the countersample surfaces is presented in Figure 7. The surface of the Ci-Ti-N and C-N countersamples are characterized by a smooth, homogeneous structure (Figure 7a,b), similar to the uncoated countersample (Figure 7d). Due to the method of fabrication, the surface of the electron-beam-melted countersample (Figure 7c) is characterized by an uneven structure resulting from the gradual solidification of the material. Unlike other types of countersamples, the C-EBM countersample surface contains many deep valleys that may constitute a reservoir of lubricant; at the same time, a surface with a large number of valleys shows a small real contact area between the asperities.

**Figure 8:**
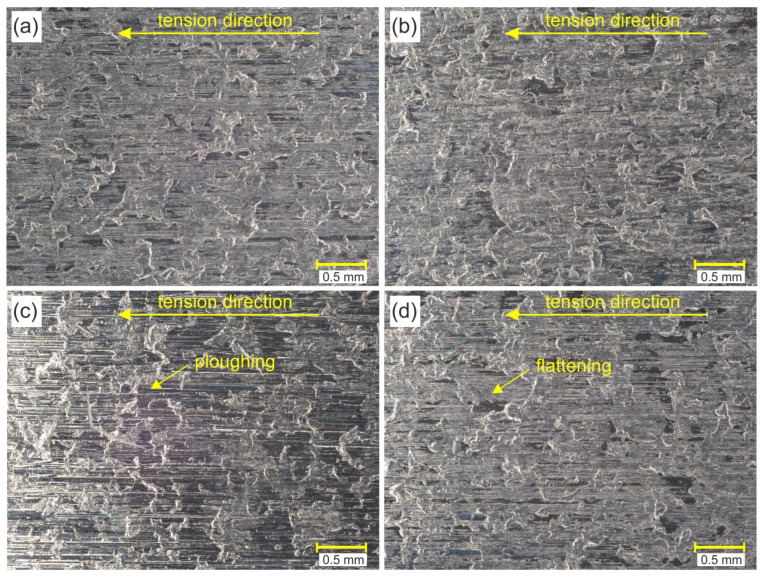
OM morphology of the sample surfaces after BUT friction test (conditions of dry friction): (**a**) C-Ti-N, (**b**) C-N, (**c**) C-EBM and (**d**) C-U.

Figure 8 shows the OM morphology of the selected surfaces of a strip sample after a friction test in conditions of dry friction. The surfaces of the sheets on the side cooperating with the surface of the countersample show a clear directional morphology of topography consistent with the tension direction. Moreover, all samples, even those tested under lubrication conditions, showed flattening of the surface asperities (Figure 8a,b,d). Friction involving the electron-beam-melted countersample was additionally accompanied by a ploughing mechanism (Figure 8c) as a result of the interaction of the hard peaks of the countersample surface with the sheet metal surface. Flattening occurred only in the area of the roughness asperities. Valleys visible on the sample surfaces were observed on samples in their as-received state. The process of stretching the samples changed their initial topography, but the valleys were not subject to direct cooperation with the surface of the countersamples.

**Figure 9:**
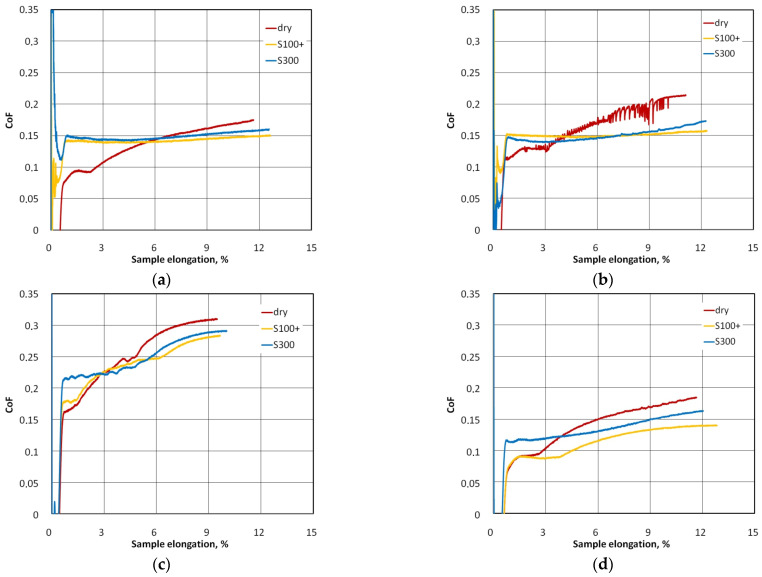
Effect of countersample coating type on the coefficient of friction: (**a**) C-Ti-N, (**b**) C-N, (**c**) C-EBM and (**d**) C-U.

Figure 9 compares the influence of friction conditions on the CoF for individual types of coatings. The difference in the influence of the type (viscosity) of lubricant on the change in the CoF value is most visible for friction involving uncoated countersamples (Figure 9d). Oil with a lower viscosity provided more favourable conditions for reducing the CoF. In the case of the remaining countersamples, the variation of the CoF for both tested oils was similar.

For the C-N countersample, clear fluctuations in the CoF are visible (Figure 9b). These instabilities under dry friction conditions may result from the stick-slip phenomenon, which causes a discrete (intermittent) movement of rubbing bodies. The reason for the stick-slip phenomenon is the difference between the coefficient of kinetic and static friction [58]. The coefficient of kinetic friction is smaller than the coefficient of static friction [59].

Countersamples C-Ti-N (Figure 9a) and C-N (Figure 9b), under lubricated conditions, provided a similar CoF value during the friction test—approximately 0.15. Within the entire range of sample elongation, the most favourable conditions for reducing the CoF were provided by uncoated samples and lubrication with S100+ oil (Figure 9d).

### 3.2. Surface Topography

**Figure 10:**
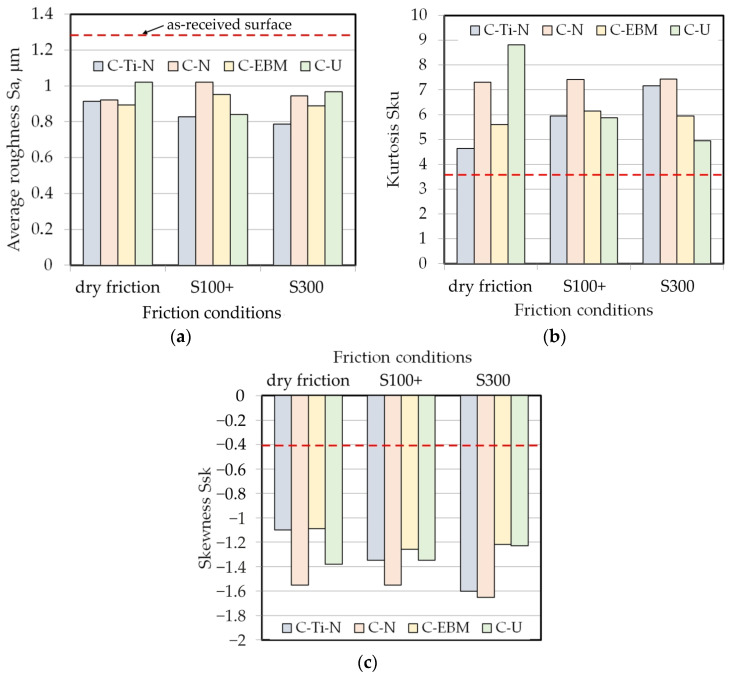
Effect of friction conditions and coating type on the friction-induced change in surface roughness parameters: (**a**) Sa, (**b**) Sku and (**c**) Ssk.

Figure 10 shows the change in the basic parameters of the sheet surface topography after the friction process. The surface roughness parameters measured on the surface of the strip samples in their as-received state were also identified as a reference. Average roughness (Sa) is the basic surface roughness parameter used to characterize the surface topography of sheet metals [60]. The results of research on design contact surfaces with reduced friction, conducted by Sedlaček et al. [61], showed that, in the case of friction in the lubrication regime, kurtosis (Sku) and skewness (Ssk) are the most suitable for describing tribological phenomena.

During the friction process, the average roughness decreased (Figure 10a) mainly as a result of the change in the topography of the sample surface caused by the elongation of the samples and the frictional contact of the interacting bodies. Under dry friction conditions, the value of the Sa parameter during the BUT test decreased by 20.3–30.2%, depending on the type of countersample. Under these conditions, countersamples coated with anti-wear coatings showed a similar change in the average roughness of the sheets and the Sa value was greater than for uncoated countersamples. When lubricated with S100+ oil with a lower viscosity than S300 oil, the average roughness decreased by 21.7–35.4%. During friction with a C-Ti-N countersample, the lowest average roughness value was obtained (Sa = 0.79 μm). Under lubricated conditions, an uncoated countersample with almost the same average roughness as the C-Ti-N countersample (Table 6) resulted in the smallest change in average roughness (Sa = 0.92 μm).

Kurtosis (Sku) is a relative measure of the concentration and flattening of the surface roughness profile [62]. If Sku > 3, the surface profile distribution curve is bell-shaped with relatively many high peaks and shallow valleys. The as-received surface and the surfaces of sheet metal after friction tests show Sku >3 (Figure 10b). Only the C-N countersample showed a similar effect on the change in skewness under all friction conditions. Dry friction involving an uncoated countersample led to the largest change in the value of kurtosis (Sku) (Figure 10b). The value of this parameter increased by 242% compared to the as-received surface. Dry friction involving a C-EBM countersample produced the smallest change in kurtosis of an as-received sheet surface. If Sku < 3, the profile elevation distribution curve is flattened and has relatively few high peaks and shallow valleys [62].

The skewness describes the asymmetry of the surface profile. The lower the Ssk value, the flatter the surface and the more rounded the summits of the surface asperities. All surfaces after the friction process showed a negative skewness value that was lower than the skewness of the as-received sheet surface. The greatest flattening of the surface asperities occurred during friction involving the C-N countersample (Figure 10c). An increase in kurtosis and skewness leads to an increase in the load-bearing coefficient and maximum contact pressure [63]. Tayebi and Polycarpou [64] found that a positive value of skewness reduces the CoF, while in the case of negative skewness the friction is more intense than for a symmetric Gaussian distribution of the surface profile. Surfaces with higher kurtosis (Sku) values and more negative skewness (Ssk) values show lower values of the CoF [65].

The analysis presented in Figure 10 is complemented by the 3D parameters shown in Table 6. As a result of the friction process, kurtosis and skewness increased and decreased, respectively, compared to the as-received sheet metal. This confirms the flattening of the surface asperities and the intensification of the friction process by increasing the contact area.

### 3.3. Temperature in the Contact Zone

**Figure 11:**
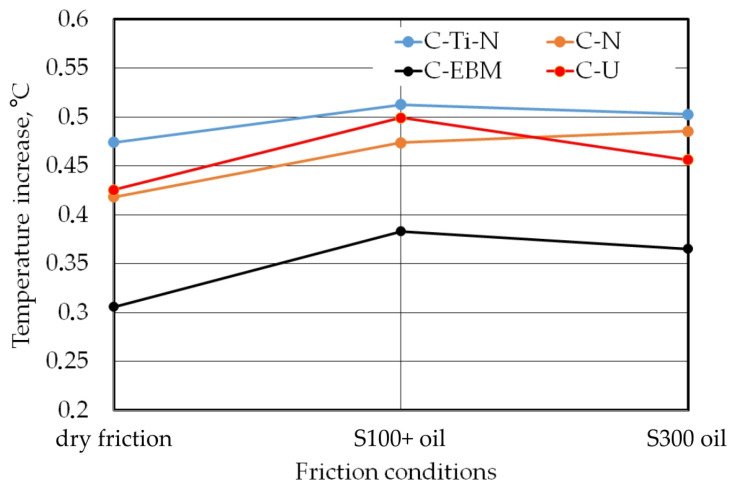
Effect of friction conditions on the change in temperature in the contact zone.

Figure 11 shows the temperature change in the contact zone. The points on this graph correspond to the maximum temperature recorded during friction tests. The increase in temperature in the contact zone is related to the internal friction of the sample material, subject to deformation and strain hardening, and external friction as a result of the frictional cooperation of the bodies of the friction pair. The process of stretching metallic samples is accompanied by an increase in temperature. As a result of the heat conduction process, heat was transferred from the sheet metal to the countersample.

Researchers usually investigate the effect of tool temperature on the value of the CoF. And it is clear that an increase in temperature in the contact zone causes an increase in the CoF value [66]. However, there are no data in the literature on the friction-induced change in temperature in the contact zone in a BUT test. This test is characterized by a relatively short friction path resulting from the elongation of the sheet metal. Nevertheless, in the BUT test, the temperature resulting from the internal friction of the stretched sample is much higher than during the strip drawing test, which is the most commonly used test for characterizing friction in deep-drawing processes.

The lowest value of temperature increase in the contact zone, under all friction conditions, was observed for the C-EBM countersample. This countersample was characterised by the highest average surface roughness (Table 6), limiting the surface area of metallic contact with the sheet surface. Due to the small metallic contact area, the heat flow between the bodies of the friction pair was limited. The remaining countersamples (C-Ti-N, C-N and C-U) facilitated the temperature increase in the contact zone due to their low average surface roughness (Table 6). For these countersamples, the temperature increase was between 0.43 and 0.53 °C.

It is also clear that the use of a lubricant increased the temperature compared to dry friction conditions. The temperature of the lubricants used corresponded to the ambient temperature. For S100+ oil and friction in the presence of countersamples C-Ti-N, C-EBM and C-U, there was a greater increase in temperature values compared to friction with S300 oil. However, the difference between the maximum temperature recorded for the C-Ti-N countersample in both lubrication conditions is very small (approximately 0.01 °C). In the case of the C-N countersample, lubrication with S300 oil led to a greater temperature increase compared to lubrication with S100+ oil. This difference should be considered as an effect of the influence of the surface topography of the countersamples and the properties of the oils (viscosity). The C-N countersample was characterized by the highest negative skewness value. If Rku < 3, the bearing area curve is flattened and has relatively few high peaks and shallow valleys. Under these conditions, there is an increased metallic contact area, which facilitates frictional heat generation. At the same time, there was a small amount of grease between the shallow valleys that could absorb heat. By filling the empty spaces between the asperities in the contact zone, the oil served as an additional agent transferring thermal energy between the rubbing bodies. Based on the results found, the impact of different lubricant viscosities on temperature change is rather marginal for the analysed coated countersamples. The difference in temperature values for both lubrication conditions is 0.0098 °C, 0.012 °C and 0.018 °C for the C-Ti-N, C-U and C-EBM countersamples, respectively. Further laboratory tests are needed in this regard.

The results of this study can help develop optimized coatings to reduce friction in the sheet metal forming process. In these processes, a hard tool comes into contact with a sheet metal with lower strength. Therefore, providing tools that reduce the CoF is crucial to ensuring long tool life and appropriate quality of the drawpiece surface. By reducing friction and wear, stamping dies may require less maintenance and experience fewer failures.

Although this article focuses on the study of friction in deep-drawing processes, the hard anti-wear coatings examined in this work may be used in toolmaking in other sheet metal forming methods, such as bending. Materials with better friction properties can improve the efficiency of forming processes by the possibility of using cheap lubricants with lower efficiency. Self-lubricating coatings are also being developed, which do not require the use of additional lubricants. These not only provide low friction, but they improve wear resistance of the tool material. The development of wear-resistant coatings for forming steel sheets is particularly important. Steel sheets and their components are widely used in the automotive industry. Any improvements in the mass production of automotive components can bring long-term economic benefits. The use of specific coatings can also meet environmental requirements by reducing the need for additional lubricants.

The knowledge gained from this study’s findings can be used to improve existing tools for sheet metal forming. However, this article only presents the results of tests using BUT friction tests. This test simulates friction conditions on the rounded edges of stamping dies. In future research, coatings will be tested using strip drawing tests and wear tests. Tribological tests will also be performed on other types of steel sheets used in the automotive industry, including high-strength sheets. This will allow for the optimal selection of coatings for a specific application and appropriate lubrication conditions.

## 4. Conclusions

This article presents the results of friction tests on DC01 steel sheets using the BUT friction test. Different lubrication conditions and types of coatings on the countersamples were tested. The investigations were focused on the influence of anti-wear coatings and lubrication conditions on the change in main surface roughness parameters and the CoF. The main conclusions can be summarized as follows:

- Under dry friction conditions, a clear increase in the CoF value, with the elongation of the samples for all analysed types of countersamples, was observed.
- Under conditions of dry friction, the highest value of the CoF, up to approximately 0.31, was recorded for the C-EBM countersample. Moreover, under lubricated conditions, the C-EBM countersample showed the highest CoF value, which is related to the much higher average roughness of this countersample compared to the other countersamples.
- Under lubricated conditions, the CoF was more stable compared to conditions of dry friction, and the uncoated countersample showed the most favourable friction conditions.
- For the uncoated countersample, oil with lower viscosity provided more favourable conditions for reducing the CoF. The most favourable conditions for reducing the CoF were provided by uncoated samples and lubrication with S100+ oil. In the case of the anti-wear coated countersamples, the variation of the CoF for both tested oils was similar.
- The average roughness decreased as a result of the friction tests, mainly as a result of the change in the topography of the sample surface caused by the elongation of the samples and the flattening mechanism of the surface asperities.
- After the friction process, kurtosis increased and skewness decreased compared to the as-received sheet metal. All surfaces after the friction process showed a negative skewness value, lower than the skewness of the as-received sheet surface. Dry friction involving an uncoated countersample led to the largest change in the value of kurtosis (Sku).
- During the friction test, a temperature change in the contact zone was observed between approximately 0.31 °C and 0.51 °C depending on the friction conditions. The lowest value of temperature increase in the contact zone, under all friction conditions, was observed for the C-EBM countersample, which is characterized as having the highest average surface roughness compared to other countersamples. High surface roughness limits the metallic contact of rubbing surfaces and, as a result, heat transfer.

## Figures and Tables

**Table 1:** Chemical composition (wt.%) of DC01 steel sheet.

C	Mn	S	P	Fe
≤0.06	≤0.35	≤0.025	≤0.025	remainder

**Table 2:** Mechanical properties of DC01 steel sheet.

Sample Orientation	Yield Stress, MPa	Ultimate Tensile Strength, MPa	Elongation, %
0°	163.4 ± 2.16	290.15 ± 1.53	37.63 ± 0.11
90°	157.7 ± 3.36	278.9 ± 0.57	35.84 ± 0.36

**Table 3:** Basic 3D surface roughness parameters of DC01 sheet surface.

Sa, μm	Sq, μm	Sz, μm	Sv, μm	Sp, μm	Sku	Ssk
1.28	1.63	13.5	8.39	5.09	3.63	−0.407

**Table 4:** Denotations and method of preparation of countersample surface.

Denotation of Countersample	Method of Preparation of Countersample Surface	Hardness, HV
C-Ti-N	Titanium- and nitrogen-ion implantation (dose: 5 × 1017 cm^−2^, accelerating voltage: 60 kV) + nitrogen-ion implantation (dose: 5 × 1017 cm^−2^, accelerating voltage: 60 kV)	305
C-N	Nitrogen-ion implantation (dose: 5 × 1017 cm^−2^, accelerating voltage: 60 kV)	306.8
C-EBM	Electron beam melting (energy density of the electron pulse: 3.13 J/cm^2^)	174.6
C-U	Uncoated	309.4

**Table 5:** Basic 2D surface roughness parameters of countersample surfaces.

Denotation of Countersample	Ra, μm	Rq, μm	Rz, μm	Rv, μm	Rp, μm	Rku	Rsk
C-Ti-N	0.03848	0.0539	0.4354	0.2816	0.1538	7.936	−0.7892
C-N	0.0705	0.1292	1.1392	0.961	0.1808	22.6	−3.384
C-EBM	1.382	1.682	7.264	3.082	4.182	2.638	0.3958
C-U	0.0363	0.0543	0.508	0.3474	0.1608	13.72	−1.402

**Table 6:** 3D surface roughness parameters of sheet metals (DF is dry friction).

Coating Type	Friction Conditions	Sp μm	Sv μm	Sz μm	Sa μm	Sq μm	Ssk	Sku
As received		5.09	8.39	13.5	1.280	1.63	−0.41	3.63
C-Ti-N	DF	2.34	7.48	9.82	0.915	1.17	−1.10	4.64
	S100+	2.41	8.04	10.5	0.827	1.09	−1.35	5.95
	S300	2.00	11.00	13.0	0.788	1.05	−1.60	7.16
C-N	DF	2.68	9.91	12.6	0.922	1.23	−1.55	7.3
	S100+	2.16	11.8	14.	1.020	1.34	−1.55	7.41
	S300	2.21	11.2	13.4	0.944	1.27	−1.65	7.44
C-EBM	DF	5.46	8.53	14.0	0.894	1.18	−1.09	5.6
	S100+	3.84	9.01	12.8	0.953	1.27	−1.26	6.14
	S300	3.94	8.62	12.6	0.889	1.18	−1.22	5.94
C-U	DF	3.03	11.50	14.5	1.020	1.38	−1.38	8.81
	S100+	2.04	9.51	11.5	0.840	1.09	−1.35	5.87
	S300	2.19	7.61	9.8	0.968	1.25	−1.23	4.95

## Data Availability

The raw data supporting the conclusions of this article will be made available by the authors on request.
